# Hunter-Gatherer Children’s Object Play and Tool Use: An Ethnohistorical Analysis

**DOI:** 10.3389/fpsyg.2022.824983

**Published:** 2022-05-11

**Authors:** Sheina Lew-Levy, Marc Malmdorf Andersen, Noa Lavi, Felix Riede

**Affiliations:** ^1^Department of Human Behavior, Ecology and Culture, Max Planck Institute for Evolutionary Anthropology, Leipzig, Germany; ^2^Department of Comparative Cultural Psychology, Max Planck Institute for Evolutionary Anthropology, Leipzig, Germany; ^3^Interacting Minds Centre, School of Culture and Society, Aarhus University, Aarhus, Denmark; ^4^Department of Anthropology, University College London, London, United Kingdom; ^5^Department of Archaeology and Heritage Studies, Aarhus University, Højbjerg, Denmark

**Keywords:** object play, tool use, hunter-gatherers (foragers), cross-cultural, childhood

## Abstract

Learning to use, make, and modify tools is key to our species’ success. Researchers have hypothesized that play with objects may have a foundational role in the ontogeny of tool use and, over evolutionary timescales, in cumulative technological innovation. Yet, there are few systematic studies investigating children’s interactions with objects outside the post-industrialized West. Here, we survey the ethnohistorical record to uncover cross-cultural trends regarding hunter-gatherer children’s use of objects during play and instrumental activities. Our dataset, consisting of 434 observations of children’s toys and tools from 54 hunter-gatherer societies, reveals several salient trends: Most objects in our dataset are used in play. Children readily manufacture their own toys, such as dolls and shelters. Most of the objects that children interact with are constructed from multiple materials. Most of the objects in our dataset are full-sized or miniature versions of adult tools, reflecting learning for adult roles. Children also engage with objects related to child culture, primarily during play. Taken together, our findings show that hunter-gatherer children grow up playing, making, and learning with objects.

## Introduction

While many species use tools (Sanz et al., 2013), human technology is unparalleled in its breadth, diversity, efficiency, and complexity. Tools have expanded our ecological and social horizons (Sterelny, 2021): traps have allowed us to reliably capture game by proxy (Trenton, 1998; Wadley, 2010); boats have given us access to new territories (Ferentinos et al., 2012; Wadley, 2013; Bjerck, 2016); and, the circulation of tools through trade and gifts has facilitated long-distance social relationships (Wiessner, 2002; Reckin and Todd, 2019). Many tools used by humans are beyond the innovative capacity of any single individual (Boyd et al., 2013). Instead, tools are improved upon through incremental modifications transmitted by cultural learning (Boyd et al., 2013; Legare and Nielsen, 2015).

Play may contribute to children’s learning about tools (Lancy, 2016; Riede et al., 2018). Play is a voluntary and spontaneous behavior that is motivating and rewarding to the individual, and which often involves repetition and iterative forms of behavior (Burghardt, 2005; Bateson and Martin, 2013). Children aged 1–5 years allocate between a quarter to a third of their time to object play, such as stacking and sorting blocks and cups (Smith, 2010; Pellegrini, 2013). As children age, objects are gradually incorporated into other forms of play, such as pretense and various games with rules (Pellegrini and Gustafson, 2005; Smith, 2010). With age, children increasingly prefer to play with visually and auditorily complex objects (Vandenberg, 1984), and object play becomes gradually more social, giving children more opportunities to observe and learn with and from others (Pellegrini, 2013).

It has been hypothesized that the repetitive nature of object play may help children safely practice the motor and cognitive skills associated with later tool use (Smith, 1982; Bjorklund and Gardiner, 2011; Solis et al., 2017). For example, Japanese infants’ play with spoons at mealtimes gradually shifts toward specific functional use with the help of mothers’ scaffolding (Nonaka and Goldfield, 2018; see also Reed, 1993). In the Okavango Delta, children’s play with objects gives them opportunities to practice grain pounding and hunting skills without risking injury or wasting resources (Bock, 2002; Bock and Johnson, 2004). Taken together, these findings support the view that play may help children build instrumental competency.

Additionally, the unconstrained and flexible nature of play has been hypothesized to contribute to the development of creative problem-solving skills (Bateson and Martin, 2013), including those required for tool innovation. Early attempts at substantiating this hypothesis found that children who played with component objects were more successful at combining them during a lure retrieval task (Sylva et al., 1976; Smith and Dutton, 1979; Pepler and Ross, 1981). These early studies were critiqued for poor controls and possible experimenter effects (Cheyne, 1982; Smith, 2010) and some subsequent studies failed to replicate their findings (Simon and Smith, 1983). Measuring naturalistic free play over the span of a school year rather than during short experimenter-led bouts, Pellegrini and Gustafson (2005) nonetheless found that children who participated in more construction play were more successful at the lure retrieval task, replicating and hence supporting findings from earlier studies. Similarly, object-oriented play was strongly associated with selecting the correct tool to retrieve a toy during an experimental task (Gredlein and Bjorklund, 2005). Recent studies also suggests that children solve instrumental problems during play by generating evidence that supports accurate causal learning (Schulz and Bonawitz, 2007; Schulz et al., 2007), discovering new object affordances (Bonawitz et al., 2009), and innovating objects using novel materials (Lew-Levy et al., 2021). Together, these findings lend support to the theoretical link between object play and some forms of creative problem solving.

Beyond the potential for learning about tool use and manufacture, object play may help children learn about their social worlds (Wynberg et al., 2021). Between 9–12 months, infants and their caregivers begin to jointly attend to objects—including during object play—through which children can learn about the intentions of others (e.g., through turn taking), develop their linguistic skills (e.g., through object naming), and learn about social norms (e.g., through object sharing) (Bakeman and Adamson, 1984; Tomasello, 1999; Tomasello et al., 2005; Mundy and Newell, 2016; Herzberg et al., 2021). The types of toys children are provisioned with may also contribute to socialization by facilitating the enactment of adult social roles (Edwards, 2000; Kollmayer et al., 2018). During their second year, children increasingly attend to the object-oriented behaviors and play of their peers (Eckerman et al., 1975). Objects and object play feature prominently in child cultures including the activities, routines, and interactions of peer groups from early childhood onward (Hardman, 1972; Corsaro and Eder, 1990).

The aforementioned research has primarily been conducted in Western post-industrial and laboratory settings. Surprisingly little is known regarding how children engage with objects in their day-to-day lives (see Herzberg et al., 2021 for exception). And, despite recent calls to diversify study populations in developmental research (Nielsen et al., 2017), ecologically valid data on play in general and object play in particular remains especially sparse for small-scale societies. Critically, descriptions of children’s everyday engagement with objects across a range of settings are needed to develop generalizable experimental play paradigms which more closely resemble children’s natural object play (Rogoff et al., 2018).

In the present paper, we draw upon the ethnohistorical record to systematically describe the types of objects used by hunter-gatherer children from a global sample of societies. Hunter-gatherer societies are small-scale societies that subsist, at least in part, on wild plants and animals. We focus on hunter-gatherers because all children in these societies must grow up to use tools to efficiently collect resources and modify their environments (Oswalt et al., 1976); because studies investigating the adaptive function of play often invoke our evolutionary history as hunter-gatherers without considering diversity in geography, cultural norms, and social histories in the past and present (Kelly, 1995; Reyes-Garciìa and Pyhaälä, 2016; Singh and Glowacki, 2021); and because our understanding of play in hunter-gatherer societies is particularly patchy (Hewlett and Boyette, 2013). The analysis presented here is exploratory; we do not aim to test existing hypotheses regarding learning through object play. Instead, we focus on developing descriptive generalizations regarding hunter-gatherer children’s engagement with objects during play and instrumental activities with the goal of improving our understanding of these behaviors across diverse cultural contexts. We view such descriptive analyses as necessary to generating new research questions regarding the form and function of object play. Building upon the limited existing literature, we specifically aim to explore five aspects of hunter-gatherer children’s engagement with objects:

(1) How do hunter-gatherer children use objects? Observational studies have found that much of hunter-gatherer children’s time is allocated to playing and working with tools (Bock, 2002; Bock and Johnson, 2004; Boyette, 2016; Crittenden, 2016; Lew-Levy et al., 2019, 2020a). Children reportedly also make their own toys (Ember and Cunnar, 2015; Crittenden, 2016). Considering this research, we investigate how interactions with objects are relatively distributed across play, instrumental activities, and toy construction.
(2) How are children’s objects constructed? Much of hunter-gatherer technology is complexly manufactured by combining multiple materials (Boyd et al., 2013; Sterelny, 2021). Here, we investigate whether this complexity is reflected in the objects children engage with.
(3) What risks do hunter-gatherer children’s objects pose? Several ethnographic studies report that hunter-gatherer children have access to, and frequently play with, potentially dangerous objects such as knives and machetes (Bakeman et al., 1990; Boyette, 2015; Crittenden, 2016; Lancy, 2016; Lew-Levy et al., 2019). Here, we systematically investigate the prevalence of risky objects used by hunter-gatherer children.
(4) In what social context do children use objects? Much of hunter-gatherer children’s play occurs with or alongside other children in the peer group (Konner, 2005; Gosso, 2010; Lew-Levy et al., 2017, 2020a). Children also learn to make toys and tools from other children and/or adults (Puri, 2013; Imamura and Akiyama, 2016; Riede et al., 2018). Considering the dense social contexts in which hunter-gatherer children grow up (Hewlett et al., 2019), we investigate the prevalence of joint engagement with objects in hunter-gatherer societies.
(5) What types of objects do hunter-gatherer children play with? Just as hunter-gatherer children’s pretense play sometimes emulates adult work (Boyette, 2019), so too do children engage with objects that emulate or are part of adult material culture (e.g., bows and digging sticks) (Nishiaki, 2013; Ember and Cunnar, 2015; Crittenden, 2016; Imamura and Akiyama, 2016; Lancy, 2016, 2017; Lew-Levy et al., 2017; Riede et al., 2018). Other objects are used by children in the playgroup only, such as games and tools used in child-only subsistence activities (Crittenden, 2016; Imamura, 2016; Gallois et al., 2017). Here, we investigate the relative proportion of children’s engagement with adult and child-only objects.

## Materials and Methods

Data was sourced from the electronic Human Relations Area Files (eHRAF^1^). The eHRAF is a searchable database of ethnographies for over 300 societies. Each paragraph is indexed following the Outline of Cultural Materials (OCM; Murdock et al., 2008), a system for classifying human behavior, beliefs, and cultural practices. Using the “Advanced Search” function, we looked for paragraphs which included information on *Technology and Material Culture* (OCM code 005), or *Games* (524) paired with *Infancy and Childhood* (850), *Socialization* (860), *Puberty and Initiation* (881), *Status of Adolescents* (882), or *Adolescent Activities* (883). We then restricted our search to societies categorized by eHRAF as hunter-gatherers or primarily hunter-gatherers (56% or more dependent on hunting, fishing, and gathering for subsistence).

**FIGURE 1:**
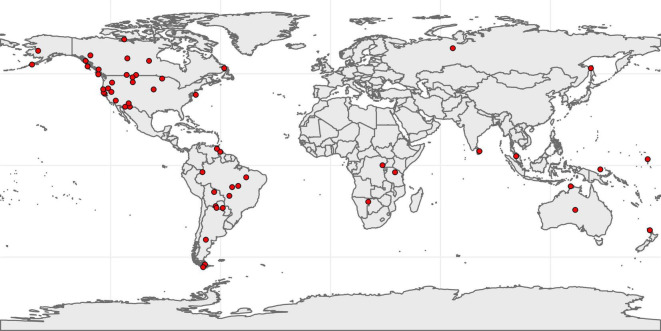
Map showing the location of the 54 societies for which data on children’s tools and toys were available. Note that 52% of societies and 60% of objects were from North America from Riede et al. (forthcoming).

Our search yielded a total of 2,285 unique paragraphs. Because our goal was to understand how children engaged with objects during play and instrumental use, we excluded paragraphs which did not contain information on children’s tools and/or toys (e.g., paragraphs which described children’s clothing, adornments, or bedding). We defined *tools* as devices or implements used to carry out specific functions, usually held in the hand. We defined *toys* as objects constructed mainly for the purpose of play. Note that these categories were not mutually exclusive; some objects were used as both toys and tools (e.g., small bows used as toys during target practice could also be used as tools to hunt birds). We excluded factory-made toys (e.g., tricycles) as these were rare and usually described in reference to formal schooling rather than subsistence and cultural activities. The resulting dataset consists of 434 toys and tools made for or by children from 54 hunter-gatherer societies (Figure 1) sourced from 272 paragraphs in 124 documents published between 1854 and 2019 (1–38 objects per society: mean count = 8.04, SD = 8.07). Each object was classified as belonging to one of eight categories (Table 1).

**TABLE 1:** Categories of objects, their definitions, and examples. Adapted from Riede et al. (forthcoming).

Category	Definition	Examples
Figure	A model of a human or of an animal, typically used as a toy	Willow horse; stuffed skin; dolls; rag babies
Game	Organized play which is structured by rules, and/or during which players coordinate their activities. Sometimes involves exercise, and/or involves feats of strength or skill	Tops; Marbles; String figures; games with balls; swings; skipping rope; kites; stilts
Musical Instrument	A device created or adapted to make musical sound	Whistles; rattles; buzz disk; bullroarer; flutes
Container	An object for holding or transporting something	Pots; bags; baskets; packs; vessels; bowls
Subsistence	*Instrument:* Hand manipulated object used in subsistence to collect relatively non-mobile or harmless food resources OR hand manipulated objects used in the manufacture of other objects. *Facility:* Form that controls the movement of a species or protects it so that it can be collected.	Knives; axes; ladder; spindle; chisel; scissors; crimper Hunting nets; fish trap; bird trap; fishing rod; lasso
Shelter	A constructed place giving permanent or temporary protection from the elements	Wickiup; hammock; hut; lodge; tent; tipi; camp
Transport	An object which conveys people or goods from one place to another	Canoe; sled; kayak; saddle; paddle
Weapon	An object designed or used for inflicting bodily harm or physical damage during hunting and/or interpersonal conflict	Bows and arrows; bolas; spears; throwing boards; blowgun; riffle; sling

To meet our aims, we used ethnographer descriptions to code additional information about the toys and tools in our dataset. Our sample of eHRAF paragraphs varies considerably in relevant information, ranging from lists of objects to detailed descriptions of objects and their contexts of use (see the Supplementary Material for examples). To maximize our sample size while minimizing subjective bias in our coding, we followed other eHRAF studies (e.g., Garfield et al., 2021) and best-practice guidelines for working with databases of this kind (Watts et al., 2022) in developing a binary coding scheme using broad and operationalizable definitions from the existing literature on children’s play and use of objects. This process resulted in five coded categories:

• **Activity.** We categorized object use as *play only* if the ethnographer explicitly mentioned play; if the activity clearly involved music, pretense, or games; if the child was using an object in a non-instrumental way; or if the child engaged in the manipulation of an object with the aim of discovering the object’s properties and attributes such as in target practice. We categorized object use as *instrumental only* if objects were exclusively described as being used in service of a goal, to access resources, or to manufacture/repair an object. If the ethnographer described the object being used both in play and instrumental activities, we coded this as *multifunctional.* Finally, we noted instances in which the *instrumental only* or *multifunctional* activities involved *toy construction.* 96% of objects in our dataset had available information regarding activity.
• **Complexity.** We categorized each object as *simple* if they were constructed with a single material, and *composite* if they were constructed with multiple materials. 76% of objects in our dataset had available information regarding their complexity.
• **Associated risk.** We categorized objects as *risky* if a child could injure themselves while using it. Risky objects included functioning boats, tools, and weapons, as well as games that involved some risk such as swings, climbing poles, and stilts. All objects in our dataset had available information regarding risk.
• **Context of use.** We categorized objects as being used *socially* if the ethnographer explicitly described multiple people simultaneously interacting with the object and each other (e.g., groups of children playing a game, children and adults setting a trap), and *solitarily* if the ethnographer explicitly stated that the object was used by a child alone. 52% of objects in our dataset had available information regarding their context of use.
• **Type.** We categorized *adult objects* as full-sized or scaled down (i.e., miniature) versions of adult tools. We identified full-sized objects by looking for reference to children borrowing objects from adults, being given functional objects by adults, or children and adults using objects together. We identified miniature tools based on ethnographer descriptions which characterized objects as “miniature,” “small” or as “toy” or “imitation” versions of adult tools. We categorized objects as *child-only objects* if these were described as exclusively children’s toys and games or used in child-only subsistence activities. All objects in our dataset had available information regarding their type.

Each category was coded by one author and reviewed by a second author. All disagreements were resolved by discussion. If relevant information needed to determine a code category was missing for an object, we coded this as “Not Available (NA).” Unless otherwise noted, supplementary analyses (Supplementary Table 1) suggest that such NA values are missing at random and thus do not bias model estimates. To assess inter-rater reliability, a student coder was trained on 10% of objects in the dataset, after which they independently coded 87 objects, representing 20% of the dataset. We calculated inter-rater reliability using Gwet’s AC1, which is less sensitive than Cohen’s Kappa to skewed distributions of categories (Feinstein and Cicchetti, 1990; Wongpakaran et al., 2013). Agreement across all categories was substantial (AC1 ≥ 0.66; Supplementary Table 2).

Using ethnographer descriptions, we also recorded the gender of the object user as *girl, boy*, or *both/unknown*. To ensure even sample sizes and reflecting developmental changes which occur between early and middle childhood (Lancy and Grove, 2011), age category for the object user was recorded as *infancy and early childhood* (approximately 6 years or younger), *middle childhood and adolescence* (approximately 7 years or older) *or age unknown*. When age was reported as a range (e.g., 6–9 years), we systematically categorized the age category according to the lower bound. If age was not reported in years (e.g., the child is described as *little* or *of early age*) we used our best judgement based on additional information available in the paragraph. For more details regarding coding scheme development and coding steps, please see Riede et al. (forthcoming).

Our data is hierarchical in structure with non-independent observations. Each row in our dataset represents an object, with multiple observations possible per paragraph, document, society, and continent. Thus, to explore our data, we fit a series of Bayesian binary logistic or multinomial multilevel regressions. All models included random effects for Paragraph, Document, and Society, which adjust estimates for imbalances in sampling across these levels (McElreath, 2015). We also included a random effect for continent, which accounts for the over-representation of North American hunter-gatherers (52% of sampled societies) inherent to eHRAF. To estimate the percent of objects per category of interest, we first follow Garfield et al. (2021) and Lightner et al. (2021) in fitting intercept-only models. To further examine our data, we then fit five additional models using the index variable approach (McElreath, 2015). Note that, to facilitate estimation in these latter models, we collapsed *play only* and *multifunctional* into a single category *any play*. Activity (Any Play = 1, Instrumental Only = 0), Complexity (Composite = 1, Simple = 0), Associated Risk (1 = Risky, Safe = 0), Context of Use (Socially = 1, Solitarily = 0), and Type (Adult Objects = 1, Child-Only Objects = 0) were each dependent variables in Models 1–5 respectively. Models 1–5 included child Gender and Age Categories as independent variables. To examine variation in the types of objects used in play vs. instrumental activities, Models 2–5 also included Activity as an independent variable.

Analyses were conducted in R Version 4.0.5 (R Core Team, 2013). Models were fit in RStan (Stan Development Team, 2022) *via* brms Version 2.16.1 (Bürkner, 2017). We specified weakly informative priors for the fixed and random effects. Each model was fit on 4 chains of 5,000 iterations, half of which were warmup iterations. All R-hat Gelman and Rubin convergence diagnostic statistics were smaller than 1.01, suggesting good mixing across all models. Results are reported with 89% Percentile Intervals (PI).

## Results

**FIGURE 2:**
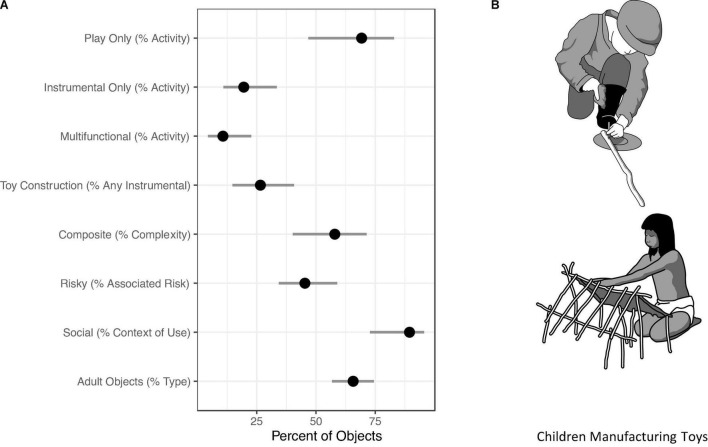
**(A)** Posterior medians estimating the percent of objects with available information for each category of interest on the probability scale, with 89% Percentile Intervals reflecting associated uncertainty. Note that the category “Any Instrumental” includes both “Instrumental Only” and “Multifunctional” **(B)** Top: A five-year-old Kaska boy making a rolling toy with the cover of a vacuum tin. Redrawn by Ea Rasmussen (Moesgård Museum) from Honigmann and Bennett (1948). Bottom: A Canela girl making a toy house just outside the village circle of houses. Redrawn by Ea Rasmussen (Moesgård Museum) from Crocker (1990).

Table 1 gives representative examples of objects included in our dataset. Table 2 reports the results of contrasts of interest from Models 1–5 as odds ratios (OR) and the proportion of the posterior above 0 (*P*_*s*_), with lower values representing “stronger certainty for a non-zero effect” (Jaeggi et al., 2021, 9). Figure 2A plots the posterior distributions for the percent of objects in our sample belonging to each category of interest. Full model results can be found in the Supplementary Material.

**TABLE 2:** Contrasts reported as odds ratios (OR) and the proportion of the posterior above 0 (*P*_*s*_).

Contrasts	Model 1 Activity		Model 2 Complexity		Model 3 Associated Risk		Model 4 Context of Use		Model 5 Type	
					
	OR	*P*_*s*_	OR	*P*_*s*_	OR	*P*_*s*_	OR	*P*_*s*_	OR	*P*_*s*_
Middle Childhood and Adolescence – Infancy and Early Childhood	2.69	0.14	1.50	0.30	1.91	0.19	1.42	0.38	1.60	0.26
Boys – Girls	1.36	0.30	**9.66**	**< 0.001**	**41.36**	**< 0.001**	1.13	0.44	**6.44**	**< 0.001**
Play – Instrumental	–	–	1.46	0.14	**0.32**	**0.001**	3.08	0.07	**0.13**	**< 0.001**

*Values in bold are those for which 89% Percentile Intervals for ORs do not cross 1.*

69% of coded objects in our database were used in play only, 20% of objects were used instrumentally only, and 11% of objects were multifunctional. 27% of objects used instrumentally (i.e., multifunctional or instrumental only) involved children manufacturing toys such as dolls, games, or play shelters (Figure 2B). Results from Model 1 (Activity) show that there were no strong differences in the gender of object users during any play vs. instrumental only activities. Contrasts reveal that children in middle childhood & adolescence were 11% more likely to participate in play with objects than those in infancy and early childhood, although predictions are uncertain (i.e., 89% PI cross zero).

57% of coded objects were composite, in the sense that they were constructed with multiple materials. Results from Model 2 (Complexity) show that there were no strong age differences in children’s use of complex vs. simple objects. Also, there was no strong difference in the use of complex objects in play vs. instrumental activities. Contrasts reveal that boys were 46% more likely than girls to use complex objects. It is important to note, however, that the gender of the user predicts missing complexity information, with less information for girls than for boys (Supplementary Table 1), suggesting a systematic pattern for missing data in this case.

45% of objects carried some risk of injury during use. Results from Model 3 (Associated Risk) suggest that boys were 69% more likely to use risky objects than girls. Risky objects were also 24% less likely to be used in play than in instrumental activities. Children in middle childhood and adolescence were 14% more likely to use risky objects than those in infancy and early childhood, although these predictions are uncertain.

89% of coded objects were used socially, in the sense that children were actively engaged with others during their use. Results from Model 4 (Context of Use) show no strong differences between social object use by age category or gender. Using objects socially was 14% more likely during play than instrumental activities, although these predictions are uncertain.

66% of objects were either full-scale or miniature versions of adult tools. Results and contrasts from Model 5 (Type) show no strong age differences in the use of full-scale and/or miniature adult tools. Boys were 74% more likely than girls to use full-scale or miniature versions of tools. Using full-scale or miniature tools was 36% less likely for play than for instrumental activities.

## Discussion

Learning to make and use tools is central to our species’ success. Many features of object play—including non-functional non-stereotypical actions, and joint social engagement—have been hypothesized to help children efficiently develop physical, cognitive, and social skills needed to make, modify, and use tools (Smith, 1982; Bjorklund and Gardiner, 2011; Bateson and Martin, 2013; Solis et al., 2017). In the present paper, we took an ethnohistorical approach to exploring object play and use in hunter-gatherer societies. In doing so, we help shed light on how objects are incorporated into the everyday lives of children outside the post-industrialized West. In what follows, we relate our findings to current research on play across cultures and discuss emerging research questions.

Most objects in our dataset were used in play. This finding echoes those from observational studies, which show that play makes up a large proportion of hunter-gatherer children’s time budgets, and that children incorporate many objects manufactured by themselves or others, as well as raw materials, into their play (e.g., Boyette, 2016; Froehle et al., 2019; Salali et al., 2019; Lew-Levy et al., 2020a). It is important to note that in some cases, children may engage in activities that are simultaneously playful and instrumental (see also Crittenden, 2016). For example, children may engage in target practice with the goal of improving their hunting skill, even if they do so during a game with peers. Similarly, children engage in instrumental activities in the service of play by making their own toys. Much of this nuance is lost in our coding scheme, partially because we have opted to use binary coding to simplify analyses, and partially because it is often not possible to identify children’s own goals *via* ethnographer descriptions. Nonetheless, these findings raise the possibility that children may be proficient at using a variety of objects to meet both playful and instrumental goals.

Children were more likely to engage with objects socially during play than during instrumental activities. Such social object play may be an important avenue for observing and imitating others, receiving teaching, learning about cultural norms such as sharing, and for innovating with peers (Bakeman et al., 1990; MacDonald, 2007; Imamura and Akiyama, 2016; Lew-Levy et al., 2017, 2020b,^2021^). Children were also more likely to use safe objects while in play than while in instrumental activities. Many ethnographers note that children in hunter-gatherer societies are free to play with dangerous objects (see Lancy, 2016 for review). Our findings support these observations: our dataset includes several examples of children engaging in play with risky objects such as knives, canoes, or stilts. Play with risky objects may have developmental benefits by helping children master age-appropriate challenges (Sandseter and Kennair, 2011). Further, play contexts may be created such that risk, including object-related risk, is minimised (Gopnik, 2020). Nonetheless, our findings suggest that children use relatively less risky objects when in play than when engaging in instrumental activities. This may be because some risky objects, such as knives and bows, are more instrumental in nature and thus, might invite more instrumental activities.

We found that many of the objects used by children were full-sized or miniature versions of adult tools, and that a majority of children’s objects were composite in nature, overall reflecting the observed complexity of adult material culture across societies (Boyd et al., 2013; Sterelny, 2021). Miniature or full-sized versions of adult objects may help children learn about adult roles and activities as well as object affordances (Riede et al., 2018). Further, adult objects were more likely to be used during instrumental than play activities, reflecting their functional nature. This finding echoes ethnographic studies which demonstrate that children learn through participation across a range of cultural contexts (Lancy, 2012; Rogoff, 2014; Lew-Levy et al., 2019). In contrast, objects reportedly used by children only such as dolls, figures, and games, were overwhelmingly used in play. Engaging with child culture artifacts during play may facilitate the acquisition of child-specific ecological knowledge (Gallois et al., 2017), the retention of technologies which have been abandoned by adults (Imamura, 2016), and the development of strong social ties with their peers (Corsaro and Eder, 1990). Some objects may facilitate learning about *both* future adult roles and peer cultures. For example, while they are not scaled-down tools, dolls nonetheless commonly represent babies. By making and playing with dolls, children may simultaneously learn about object affordances, practice adult social roles, and reinterpret adult culture to meet the concerns of their peer world (Corsaro, 1993; Edwards, 2000).

There were no gender differences in objects used in play vs. instrumental activities, reflecting findings from time allocation studies on the topic in hunter-gatherer societies (Boyette, 2016; Lew-Levy et al., 2020a). Boys were more likely to use risky objects, which may echo cross-cultural findings regarding gender differences in risk taking (Apicella et al., 2017). Our results regarding age were imprecise and hence inconclusive, largely because few ethnographers provided enough detail to confidently attribute age categories to object users, resulting in most user ages being categorized as “unknown.” Tentatively, however, our results suggest that children in middle childhood and adolescence (i.e., seven years or over) were more likely to use objects during play and more likely than infants and children in early childhood (i.e., six years or younger) to use risky objects, suggesting that children’s use of objects becomes more varied with age. Note that the increased use of risky objects with age need not signify actual increased risk in object use; this could be an expression of older children having acquired the necessary skill to wield risky objects safely.

Our study has several limitations. We had few observations for precise age categories, limiting our ability to infer developmental trends in object play and use. In the case of object complexity, values were not missing completely at random, but instead are biased toward missing values for girls. Gender differences related to complexity should thus be interpreted cautiously. By focusing on inanimate objects, we have overlooked how children play with babies and animals, the form and learning function of which may share similarities with some forms of object play. The records included within eHRAF reflect biases inherent to the ethnohistoric literature: virtually all ethnographers were adults, and most were men. In addition, all observations were made before the full advent of interest in children as culture-bearers and prior to the emergence of systematic studies in this domain. As a result, many aspects of children’s activities may be less systematically recorded compared to other aspects of culture. In addition, eHRAF is known for its bias toward North America. While it does represent the single best source for comparative cross-cultural analysis, and while we used statistical methods to overcome such biases, the sample’s representativity in the strict sense cannot be claimed. Finally, the present study used binary coding of variables of interest to facilitate analysis and because ethnographer descriptions often lacked the details necessary for more continuous coding (e.g., ratings). We acknowledge that this approach obscures much of the nuance inherent to children’s activities, and indeed, human behavior more generally.

Despite these caveats, our descriptive study points to several new avenues of research which can help further our understanding regarding the learning function of object play across individuals and societies. First, many experimental studies examine how children’s play with raw materials (e.g., clamps, sticks, pipe cleaners) contributes to their ability to modify and recombine these into functional tools. However, these tasks often represent ill-structured problems in the sense that children lack information about the transformations needed to accomplish the desired end goal (Cutting et al., 2011). Our data suggests that in contrast to playing with component pieces, hunter-gatherer children often play with composite objects. By engaging with the end-state first, children may more easily come to understand the functional properties of component pieces, and thus, may more easily apply their knowledge to tool selection and modification tasks (Cutting et al., 2014; Riede et al., 2018). Some experimental studies investigating the learning function for play also focus on children’s solo play with objects. However, our findings suggest that most play with objects occurs socially. Developmental research has long demonstrated that collaborative learning bolsters children’s ability to solve novel tasks (Azmitia, 1988; Perlmutter et al., 1989; Rendell et al., 2011) and their logical reasoning skills (Tomasello et al., 1993; Kruger and Tomasello, 1996). Such socio-cognitive capabilities may also be central to children’s ability to make and innovate tools (Gönül et al., 2019; Lew-Levy et al., 2021). Next, most experimental research on object play focuses on deferred functions related to tool use and tool making skill. However, our findings hint at the possibility that playing with objects may more immediately have a central role in the development and maintenance of peer cultures. Finally, if object play contributes to the development of problem solving skills, then the diversity and complexity of children’s play objects should covary with that of adult toolkits (Riede et al., 2018). Testing this possibility requires careful attention to potential confounds such as environmental risk, population size, raw material availability, and subsistence strategy (Kline and Boyd, 2010; Collard et al., 2013). We are in the process of expanding our dataset to include these variables in the hopes of further investigating how children’s learning through object play contributes to the observed cross-cultural variation in material culture. Such analyses will help shed new light on how object play and play object provisioning may have bolstered technological innovation in the past.

## Data Availability Statement

The datasets presented in this study can be found in online repositories. The names of the repository/repositories and accession number(s) can be found below: https://github.com/sheinalewlevy/Toys-As-Teachers/tree/main/Frontiers.

## Author Contributions

SLL designed the study, analyzed the data, and wrote the manuscript. SLL, MMA, NL, and FR secured funding for the project, developed the dataset, and coded the data. MMA, NL, and FR critically revised the manuscript. All authors contributed to the article and approved the submitted version.

## Conflict of Interest

The authors declare that the research was conducted in the absence of any commercial or financial relationships that could be construed as a potential conflict of interest.

## Publisher’s Note

All claims expressed in this article are solely those of the authors and do not necessarily represent those of their affiliated organizations, or those of the publisher, the editors and the reviewers. Any product that may be evaluated in this article, or claim that may be made by its manufacturer, is not guaranteed or endorsed by the publisher.

## Footnotes

1
https://ehrafworldcultures.yale.edu/ehrafe/
