# Supplementary material for: Hunter-Gatherer Children’s Object Play and Tool Use: An Ethnohistorical Analysis
Source: Front Psychol. 2022 May 11;13:824983. doi: 10.3389/fpsyg.2022.824983 (PMC9132165; doi:10.3389/fpsyg.2022.824983)
Supplement: Supplementary file 1 [file Data_Sheet_1.pdf]

Supplementary Material

1 Examples of paragraphs

In Riede et al. *"Toys as teachers: A cross-cultural analysis of object play and enskillment in hunter-gatherer societies"* (in revision at Journal of Archaeological Method and Theory) we describe the varying levels of detail available on children's toys and tools as follows:

The detail with which children's toys and tools were described varied considerably by ethnographer and surveyed community. For example, Pearsall (1950, p. 343) describes the materials, transmission, and developmental processes by which Klamath boys learn to make and use bows:

At first they play with a tiny bow of willow, little more than a toy, which they learn to make from an older boy or from their father. But by the time a boy is six or seven his father presents him with a real bow made from juniper. It is smaller than the large yew war and hunting bows of the men, and the arrows are blunt. The boys practice shooting at targets around the camp. They will not begin to do any hunting for several years, but groups of boys imitate the shooting contests of their elders.

Seligman et al. (1931, pp. 91–92) describe how Vedda children manufacture ladders while emulating honey collecting during play, and how community members participated in this game:

One thing is taught the lads systematically, that is the method of collecting honey from the combs of the rock bee. Whenever the caves are conveniently situated a ladder of creepers is suspended from a tree in the jungle above and hangs over the end of the face of rock which forms the cave. On this the youths play at "honey getting." At Pihilegodagalge the lads were quite willing to demonstrate to us how it was done, and the elder men showed clearly that this was a game which they encouraged. A lad of about thirteen collected some green leaves and tied them together with creeper, then taking an arrow, a toy masliya, and a broken gourd tied with creeper, which hung over his arm, for a maludema [a deerskin vessel for honey-collecting], he set fire to the leaves and climbed the ladder. While lowering the smoker and letting the smoke blow into the crevice in the rock where the comb was supposed to be, he pretended to cut round its sides with an arrow and thrust at it with his masliya [a four-pronged implement used in honey-collecting], from which he transferred the honey into the gourd. As he descended from the ladder he beat his chest and sides as though driving off the bees, and directly he reached the ground rushed into the jungle to escape from them, all the smaller children imitating him with great glee. Obviously this was a well-known and favourite game, for even the elders took part in it, throwing their clothes over their heads and running into the jungle.

At the other end of the spectrum, ethnographers simply list the objects children played with. For example, Nimuendajú (1948, p. 718) states of Ticana children:

They play with dolls carved of muirapiranga, figures of animals, little canoes, small bows and arrows, and buzz-disks of gourd shells.

Many ethnographers allude to how objects feature in children's knowledge acquisition, without providing further detail about how and from whom children received the objects. Among the Tlingit, Emmons and De Laguna (1991, p. 101) state that:

[Children] were taught to pack by carrying a light pack about every day for an hour or so.

2 Supplementary Tables

Table S1. Coefficients (β) and Standard Deviations (SD) on the log odds scale from multilevel binary logistic regressions examining whether individual characteristics (Gender and Age Category) predict missing values. Values in bold represent coefficients for which 89% Percentile Intervals cross zero. We considered strong differences between known categories (girls vs. boys, middle childhood and adolescence vs. infancy and early childhood) as suggesting that there is systematic bias for missing values in our data. Random Effect SDs are in Table S5.

	Model S1 (Activity)		Model S2 (Complexity)		Model S3 (Social Context)		Model S4 (Age Category)	
	β	SD	β	SD	β	SD	β	SD
Intercept	**-7.01**	**1.99**	-0.33	0.53	-0.56	0.95	**-12.35**	**6.40**
Boys	-0.51	0.75	**-1.22**	**0.37**	-0.64	0.55	-0.02	0.97
Both Genders/Unknown	-0.09	0.74	**-0.76**	**0.38**	**-1.00**	**0.59**	0.01	0.99
Middle Childhood and Adolescence	-0.24	0.95	0.26	0.59	-0.62	0.80	--	--
Age Unknown	0.37	0.84	**-0.80**	**0.44**	0.19	0.68	--	--

Reference value for Gender: Girls. Reference value for Age Category: Infancy and Early Childhood.

Table S2. Percent agreement and Gwet's AC1 statistic for variables of interest. Based on inter-coding for 20% of the dataset (87 objects).

Variable	Percent agreement	Gwet's AC1	95% Confidence Intervals
Any play	81.6	0.84	0.73, 0.95
Multifunctional	79.3	0.79	0.67, 0.91
Toy construction	83.9	0.94	0.91, 0.97
Complexity	80.5	0.66	0.48, 0.84
Risk	92.0	0.84	0.73, 0.96
Context	69.0	0.68	0.53, 0.83
Type	93.1	0.92	0.84, 1

Table S3. Coefficients (β) and Standard Deviations (SD) on the log odds scale from intercept-only multilevel multinomial (Model S5) and binary logistic (Models S6-S10) regressions estimating the percent of objects per category of interest. Values in bold represent coefficients for which 89% Percentile Intervals cross zero. Random Effect SDs are in Table S5.

	Model S5				Model S6		Model S7		Model S8		Model S9		Model S10	
	Play		Multifunctional		Toy Construction		Complex		Risky		Social		Type	
	β	SD	β	SD	β	SD	β	SD	β	SD	β	SD	β	SD
Intercept	**1.23**	**0.52**	-0.62	0.44	**-1.03**	**0.45**	0.28	0.42	-0.18	0.32	**2.10**	**0.67**	**0.66**	**0.26**
Subset of Observations	All				Any Instrumental		All		All		All		All	
Number of Observations	*416*				*185*		*330*		*434*		*227*		*434*	

For Model S5 (Activity) the reference category was *instrumental only.*

Table S4. Coefficients (β) and Standard Deviations (SD) on the log odds scale from the multilevel binary logistic regressions using the index variable approach (McElreath, 2015). Values in bold represent coefficients for which 89% Percentile Intervals do not cross zero. Random Effect SDs are in Table S5.

	Model 1 (Activity)		Model 2 (Complexity)		Model 3 (Associated Risk)		Model 4 (Context of Use)		Model 5 (Type)	
	β	SD	β	SD	β	SD	β	SD	β	SD
Intercept	**1.36**	**0.77**	-0.01	0.76	0.03	0.73	0.85	0.82	0.81	0.73
Infancy and Early Childhood	-0.14	0.75	-0.21	0.65	-0.11	0.66	-0.31	0.84	0.30	0.65
Middle Childhood and Adolescence	0.84	0.75	0.22	0.69	0.54	0.66	0.07	0.81	0.75	0.68
Age Unknown	0.65	0.65	0.00	0.54	-0.40	0.59	1.09	0.71	-0.23	0.59
Girls	-0.26	0.64	-0.83	0.57	**-1.80**	**0.62**	0.20	0.74	-0.40	0.57
Boys	0.06	0.62	**1.45**	**0.58**	**1.94**	**0.59**	0.33	0.70	**1.48**	**0.58**
Both Genders/Unknown	**1.57**	**0.65**	-0.65	0.55	-0.12	0.58	0.32	0.71	-0.26	0.56
Instrumental Activities	--	--	-0.16	0.58	0.39	0.62	0.16	0.75	**1.22**	**0.62**
Play Activities	--	--	0.23	0.60	-0.73	0.60	**1.29**	**0.72**	-0.82	0.58
Unknown Activities	--	--	-0.11	0.75	0.40	0.75	-0.65	0.94	0.36	0.74
Number of Observations	*416*		*330*		*434*		*227*		*434*	

Table S5. Random Effect Standard Deviations for Models 1-5 and S1-S10.

	1	2	3	4	5	S1	S2	S3	S4	S5P	S5M	S6	S7	S8	S9	S10
Continent	1.00	0.92	0.43	0.60	0.29	1.19	0.28	0.54	0.90	0.83	0.41	0.49	0.83	0.37	0.77	0.25
Paragraph	3.57	0.69	0.75	2.81	0.86	1.84	1.10	4.96	0.76	3.27	1.35	1.34	0.59	1.19	2.44	0.91
Source Text	0.69	0.43	1.01	1.39	0.76	1.54	0.49	1.41	0.78	0.62	0.47	0.77	0.37	1.21	1.07	0.82
Society	0.49	0.30	0.74	1.25	0.37	1.82	0.68	0.62	0.79	0.57	0.63	0.91	0.25	0.55	1.02	0.40

Model S5 was a categorical model, P=*Play Only*, M=*Multifunctional*, reference=*Instrumental Only*.

3 Supplementary Figures

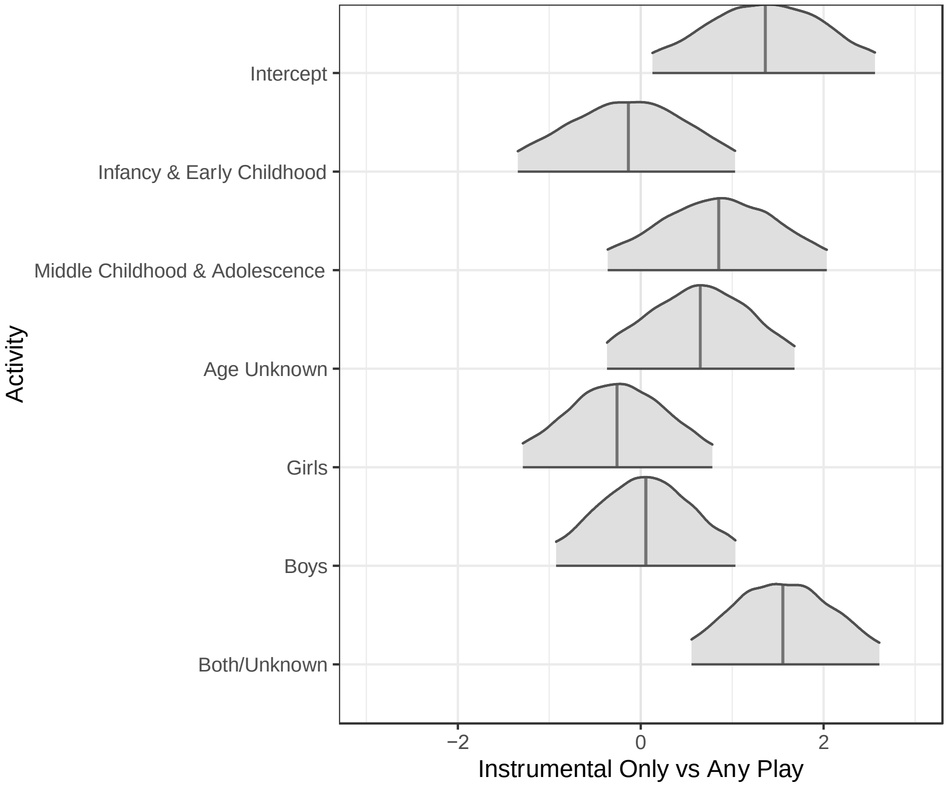

Figure S1. 89% Percentile Interval density plot with mid-lines representing the median values on the log odds scale. Where most of the distribution falls above or below zero, the variable is predicted to have a stronger effect. Negative values reflect greater probability for instrumental only, and positive values reflect greater probability for any play.

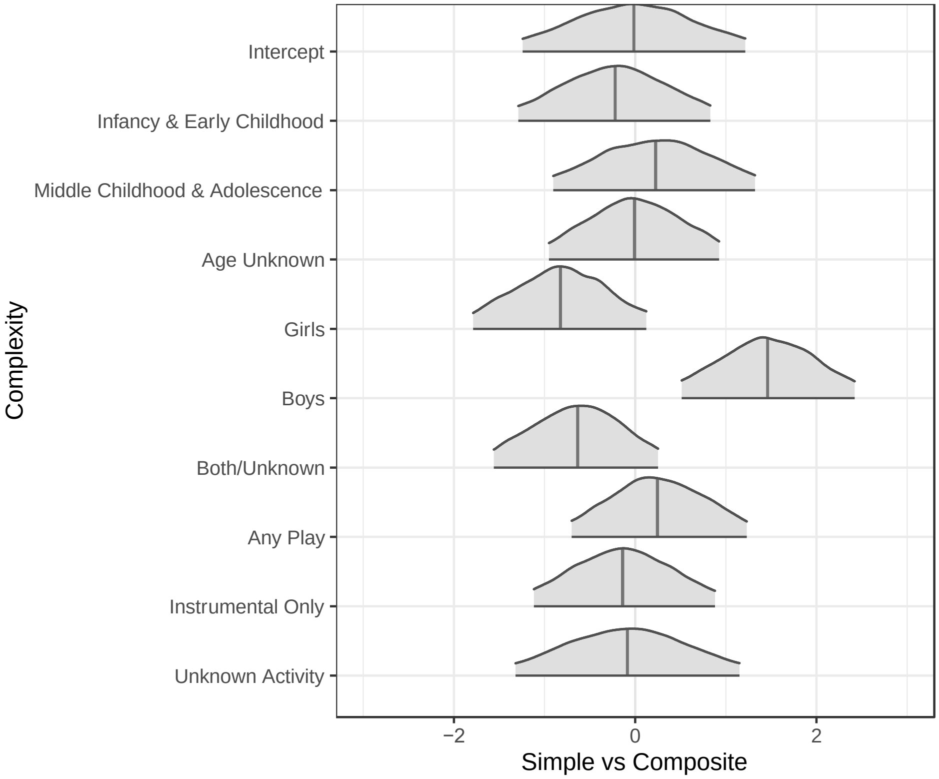

Figure S2. 89% Percentile Interval density plot with mid-lines representing the median values on the log odds scale. Where most of the distribution falls above or below zero, the variable is predicted to have a stronger effect. Negative values reflect greater probability for simple objects, and positive values reflect greater probability for composite objects.

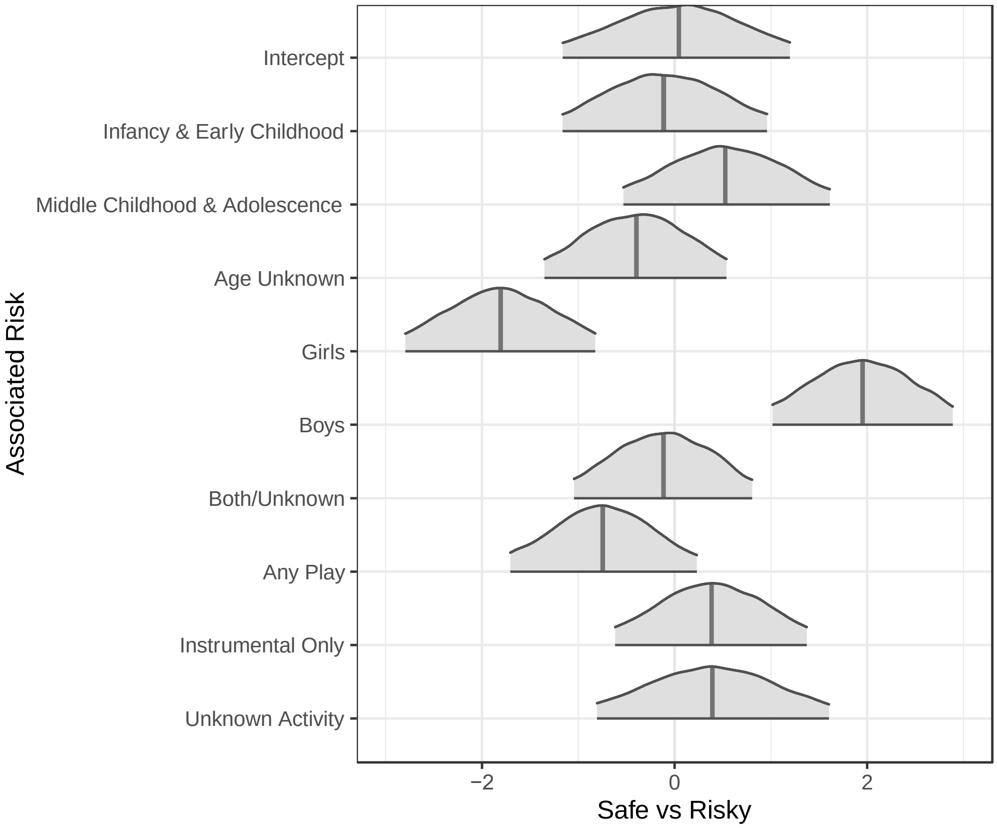

Figure S3. 89% Percentile Interval density plot with mid-lines representing the median values on the log odds scale. Where most of the distribution falls above or below zero, the variable is predicted to have a stronger effect. Negative values reflect greater probability for safe objects, and positive values reflect greater probability for risky objects.

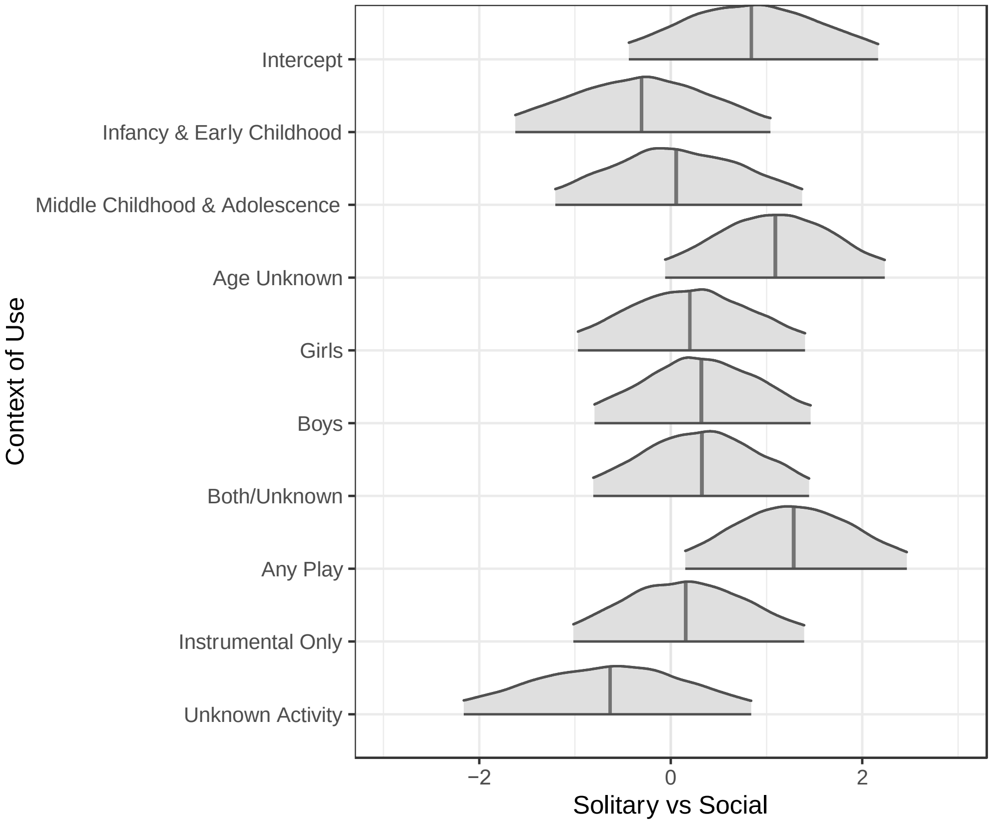

Figure S4. 89% Percentile Interval density plot with mid-lines representing the median values on the log odds scale. Where most of the distribution falls above or below zero, the variable is predicted to have a stronger effect. Negative values reflect greater probability for objects used solitarily, and positive values reflect greater probability for objects used socially.

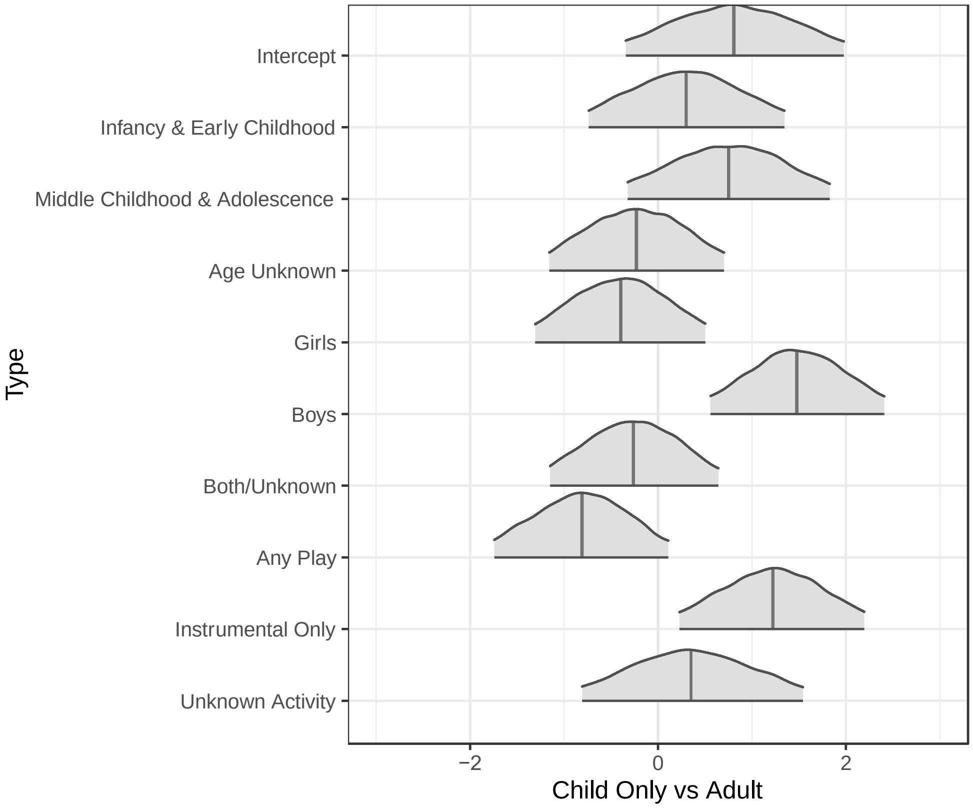

Figure S5. 89% Percentile Interval density plot with mid-lines representing the median values on the log odds scale. Where most of the distribution falls above or below zero, the variable is predicted to have a stronger effect. Negative values reflect greater probability for child only objects, and positive values reflect greater probability for adult objects.
